# Resting-State Brain Functional Hyper-Network Construction Based on Elastic Net and Group Lasso Methods

**DOI:** 10.3389/fninf.2018.00025

**Published:** 2018-05-15

**Authors:** Hao Guo, Yao Li, Yong Xu, Yanyi Jin, Jie Xiang, Junjie Chen

**Affiliations:** ^1^Department of Software Engineering, College of Information and Computer, Taiyuan University of Technology, Taiyuan, China; ^2^National Laboratory of Pattern Recognition, Institute of Automation, The Chinese Academy of Sciences, Beijing, China; ^3^Department of Psychiatry, First Hospital of Shanxi Medical University, Taiyuan, China

**Keywords:** depression, hyper-network, elastic net, group lasso, classification

## Abstract

Brain network analysis has been widely applied in neuroimaging studies. A hyper-network construction method was previously proposed to characterize the high-order relationships among multiple brain regions, where every edge is connected to more than two brain regions and can be represented by a hyper-graph. A brain functional hyper-network is constructed by a sparse linear regression model using resting-state functional magnetic resonance imaging (fMRI) time series, which in previous studies has been solved by the lasso method. Despite its successful application in many studies, the lasso method has some limitations, including an inability to explain the grouping effect. That is, using the lasso method may cause relevant brain regions be missed in selecting related regions. Ideally, a hyper-edge construction method should be able to select interacting brain regions as accurately as possible. To solve this problem, we took into account the grouping effect among brain regions and proposed two methods to improve the construction of the hyper-network: the elastic net and the group lasso. The three methods were applied to the construction of functional hyper-networks in depressed patients and normal controls. The results showed structural differences among the hyper-networks constructed by the three methods. The hyper-network structure obtained by the lasso was similar to that obtained by the elastic net method but very different from that obtained by the group lasso. The classification results indicated that the elastic net method achieved better classification results than the lasso method with the two proposed methods of hyper-network construction. The elastic net method can effectively solve the grouping effect and achieve better classification performance.

## Introduction

Evidence from numerous anatomical and physiological studies suggests that cognitive processing depends on the interaction among distributed brain regions (Sporns, 2014). A brain functional network is a simplified representation of brain interactions and has been widely applied in studies of mental disorders, including epilepsy (Zhang et al., 2012), major depressive disorder (MDD) (Kaiser et al., 2016), Alzheimer's disease (Pievani et al., 2011), and schizophrenia (Lynall et al., 2010). The rapid development of neuroimaging technology provides a good foundation for research on brain functional networks. In recent years, the use of resting-state functional magnetic resonance imaging (fMRI) for mapping neural functional networks has attracted increasing attention. A low frequency blood oxygen level-dependent (BOLD) signal is associated with spontaneous neuronal activity in the brain (Zeng et al., 2012), and the interaction among brain regions in a resting state can be represented by the functional network constructed by the BOLD signal.

Various analytical methods have been proposed for modeling brain functional connectivity from fMRI data, including correlation (Bullmore and Sporns, 2009; Sporns, 2011; Wee et al., 2012; Jie et al., 2014), graphical modeling (Bullmore et al., 2000; Chen and Herskovits, 2007), partial correlation (Salvador et al., 2005; Marrelec et al., 2006, 2007), and sparse representation methods (Lee et al., 2011; Wee et al., 2014). The correlation method is the most common and has been successfully applied to the classification of patients and normal controls (Zeng et al., 2012; Ye et al., 2015). However, one of its main limitations is that it can only capture pairwise information and therefore cannot fully reflect the interactions among multiple brain regions (Huang et al., 2010). Moreover, a network based on correlations may include a number of false connections due to the arbitrary selection of thresholds (Wee et al., 2014). The other methods also have their shortcomings. When used to study brain connections, graphical models lack prior knowledge (Huang et al., 2010), such as which brain regions should be involved and how they are connected. Partial correlation estimation is usually achieved using the maximum likelihood estimation (MLE) of the inverse covariance matrix. However, a limitation of this method is that the data sample size required for a reliable estimate is much larger than the number of modeled brain regions (Huang et al., 2010). Sparse inverse covariance estimation (Zhou et al., 2014; Fu et al., 2015) resolves the deficiencies of MLE to some extent, but there are still problems with this approach. Although it is effective for learning sparse connection networks, it is not suitable for evaluating the connections due to shrinkage effects (Smith et al., 2011). Sparse representation can filter out false or insignificant connections by applying regularization parameters to produce sparse networks. However, apart from sparse structures, brain functional networks usually include other types, such as small world, scale-free topology, hierarchical, and modular structures (Sporns, 2010). Wee et al. (2014) adopted the group lasso method using l_2,1_ regularization for functional connectivity modeling to estimate networks with the same topology but different connection strengths, while ignoring the network topology patterns of specific groups.

The traditional methods describe the relationship between two regions. However, later studies indicate that interactions take place not only between two regions, but among multiple regions. Recent neuroscience studies have identified significant higher-order interactions in neuronal spiking, local field potentials, and cortical activities (Montani et al., 2009; Ohiorhenuan et al., 2010; Yu et al., 2011). In particular, studies suggest that one brain region predominantly interacts directly with a few other brain regions in neurological processes (Huang et al., 2010). Functional networks based on pairwise relationships can thus only reflect the second-order relationships between brain regions, ignoring the high-order relationships that may be crucial for studies of underlying pathology.

The hyper-network (Jie et al., 2016) method was proposed to address this issue. Hyper-networks (Jie et al., 2016) are based on hyper-graph theory. Each node represents a brain region and each hyper-edge includes many nodes that represent the interactions among multiple brain regions. The existing method of constructing brain functional hyper-network is to use sparse regression model. According to the model, the sparse solution could be produced, and the nonzero elements in sparse solution represent correlation. Using the sparse linear regression model, a region can be represented as a linear combination of other regions and its interactions with a few other regions can be obtained. Insignificant and false interactions are forced to zero. In hyper-network construction, the process of obtaining sparse solution is solved by the least absolute shrinkage and selection operator (lasso) method (Jie et al., 2016). However, the limitation of using the lasso method to solve the sparse linear regression model is that when constructing the hyper-edges of a designated brain region, if the pairwise correlations among other brain regions are very high, then the lasso tends to select only one region from the group with a grouping effect (Zou and Trevor, 2005). As this may mean that some related areas cannot be selected, the method lacks the ability to explain the grouping information.

To solve the problem of the grouping effect among brain regions, we propose two alternative methods to improve the construction of a hyper-network: (1) the elastic net (De Mol et al., 2008; Furqan and Siyal, 2016; Teipel et al., 2017) and (2) the group lasso method (Friedman et al., 2010a; Yu et al., 2015; Souly and Shah, 2016). Then we extracted features using the different clustering coefficients defined by hyper-network to depict the functional brain network topology and performed non-parametric test to select those features with significant difference. Finally, we applied a multi-kernel support vector machine (SVM) technique on the selected features for classification. Besides, we analyzed network topology based on three methods using hyper-edges and average clustering coefficients. Furthermore, the comparative analysis of depressed patients was performed via using the classification features with significant differences between groups. The classification results showed that the elastic net method achieved better classification results than the lasso method. In addition, we further analyzed the influence of the model parameters and the classifier parameters.

## Materials and methods

**Figure 1:**
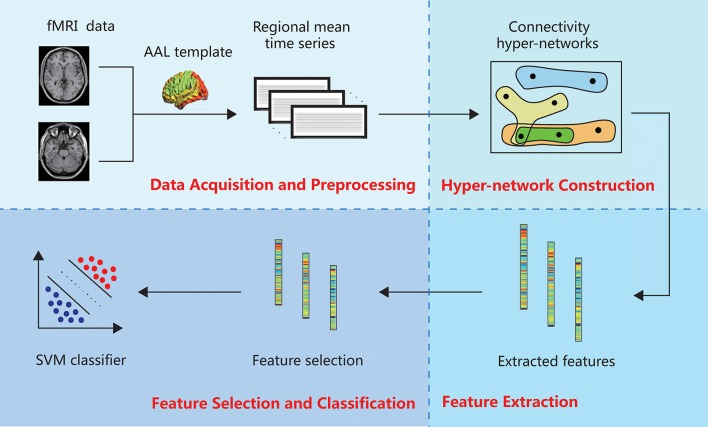
Framework of brain network classification method based on hyper-network.

The hyper-network method of brain network classification involves data acquisition and preprocessing, construction of the hyper-network, feature extraction, feature selection and classification. In this section, we describe each of these steps in detail. Figure 1 illustrates the whole process.

### Data acquisition and preprocessing

Before starting the study, written agreement was obtained from each participant in accordance with the recommendations of the Shanxi Medical Ethics Committee (reference number: 2012013). All of the participants signed written informed consent according to the Helsinki Declaration. Seventy subjects were recruited: 38 first-episode, drug-naive patients with MDD (15 male; mean age 28.4 ± 9.68 years, range 17–49) and 28 healthy right-handed volunteers (13 male; mean age 26.6 ± 9.4 years, range 17–51). Data from four participants were discarded due to problems with the data. Resting-state fMRI was performed on all subjects using a 3T magnetic resonance scanner (Siemens Trio 3-Tesla scanner, Siemens, Erlangen, Germany). The detailed demographics and Clinical Characteristics of the Subjects were illustrated by Table 1.

**Table 1:** Demographics and Clinical Characteristics of the Subjects.

	**CON**	**MDD**	***P*-value**
Age(years)	17–51 (26.6 ± 9.4)	17–49 (28.4 ± 9.68)	0.44^a^
Sex (male/female)	13/15	15/23	0.57^b^
Handedness (R/L)	28/0	38/0	–
HAMD	N/A	15–42 (22.8 ± 13.3)	–

*Data are presented as the range of minimum to maximum (mean ± SD). HAMD, Hamilton Depression Rating Scale; MDD, major depressive disorder; NA, nonapplicable; CON, normal controls*.

a *The P-values were obtained by two-sample two-tailed t-test*.

b *The P-value were obtained by two-tailed Pearsons χ ^2^-test*.

Data acquisition was completed at the First Hospital of Shanxi Medical University. All scans were performed by radiologists familiar with magnetic resonance imaging. During the scan, subjects were asked to stay awake, and to relax and close their eyes while not thinking about anything in particular. Each scan consisted of 248 contiguous EPI functional volumes with the scan parameters set as follows: axial slices = 33, repetition time = 2,000 ms, echo time = 30 ms, thickness/skip = 4/0 mm, field of view = 192 × 192 mm, matrix = 64 × 64 mm, flip angle = 90°. Due to the instability of the initial magnetic resonance signal and the adaptability of the subjects to the environment, the time series of the first 10 functional volumes were discarded. The detailed scanning parameters are provided in the Supplemental Text S1.

Functional data preprocessing was performed using the statistical parametric mapping (SPM8) software package (http://www.fil.ion.ucl.ac.uk/spm). First, the datasets were corrected for slice timing and head motion. Two samples from depressed patients and two from controls that exhibited more than 3.0 mm of translation and 3 degrees of rotation were discarded, and thus were not included in the 66 samples for data analysis. The corrected images were optimized using 12-dimensional affine transformation and spatially normalized to 3 × 3 × 3 mm voxels in the Montreal Neurological Institute standard space. Finally, linear detrending and band-pass filtering (0.01–0.10 Hz) were performed to reduce the effects of low-frequency drift and high-frequency physiological noise.

### Hyper-graph theory

**Figure 2:**
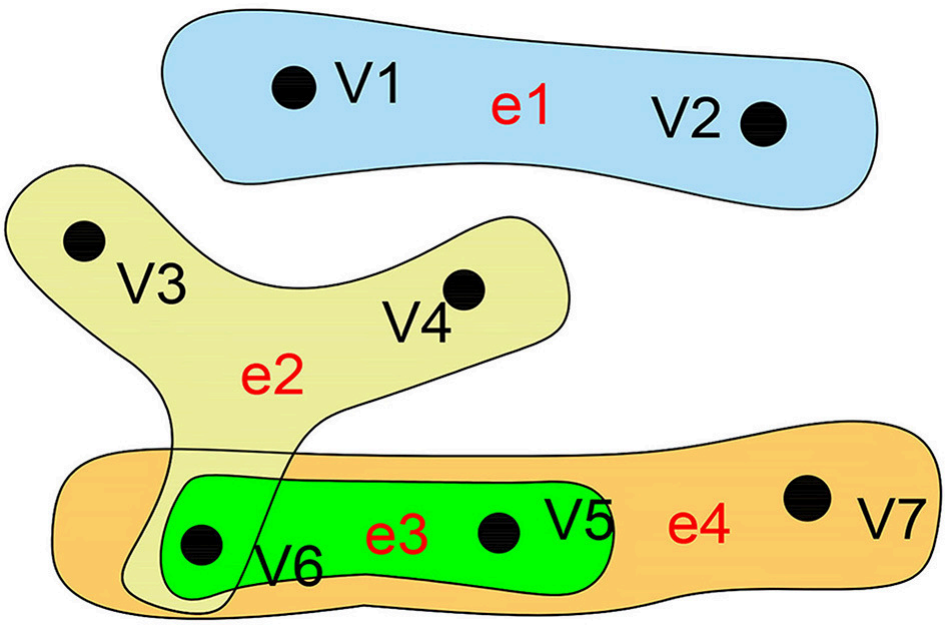
Hyper-graph. A hyper-graph in which each hyper-edge can connect more than two nodes. Here, the hyper-graph contains 7 nodes and 4 edges. V = {v_1_,v_2_,v_3_,v_4_,v_5_,v_6_,v_7_}, E = {e_1_,e_2_,e_3_,e_4_}, e_1_ = {v_1_,v_2_}, e_2_ = {v_3_,v_4_,v_6_}, e_3_ = {v_5_,v_6_},e_4_ = {v_5_,v_6_,v_7_}.

As a branch of mathematics, graph theory has been widely applied to analyze the functional interaction between brain regions, mainly by unambiguously discretizing the brain into distinct nodes and their interconnecting edges (Fornito et al., 2013). A traditional graph only characterizes two correlated nodes but ignores the high-order information, which can be represented by a hyper-graph. The biggest difference between a hyper-graph and a traditional graph is that one hyper-edge of the hyper-graph can connect to more than two nodes. Compared with the traditional graph, the hyper-graph pays more attention to the relationships than to the nodes. In short, the hyper-edge contains an unfixed number of points, which means it is a kind of intuitive mathematical expression reflecting multivariate relational data. Hyper-graph s are widely applied in many fields of computer science (Mäkinen, 1990), especially the Internet of Things, social networks, large-scale integrated design, relational databases, biomedicine, and many other applications where there are complex associations among large numbers of non-independent data points. Researchers are increasingly finding that multivariate relationships can more naturally express the internal relations and patterns hiding in information. In previous similar studies, hyper-graph s have been successfully applied to image classification (Yu et al., 2012), protein function prediction (Gallagher and Goldberg, 2013), and pattern recognition (Ren et al., 2011). Figure 2 illustrates an example of a hyper-graph.

The mathematical expression of a hyper-graph can be represented by *H* = *(V,E)* (Kaufmann et al., 2009), where *V* represents a set of nodes and *E* represents a set of hyper-edges, and hyper-edge *e* ∈ *E* is a subset of *V*. If any two hyper-edges of a hyper-graph are not contained in each other, then the hyper-graph is called an irreducible or simple hyper-graph (Berge, 1989). We can use a |*V*| × |*E*| incidence matrix to represent *H*:

(1) $$H(v,e)=\{\begin{array}{c} 1,\;if\;v\in e\\ 0,\;if\;v\notin e \end{array}$$

*H*(*v, e*) represents the corresponding element in the incidence matrix, *v* ∈ *V* represents the node, and *e* ∈ *E* represents the hyper-edge. Nodes are the column elements of the incidence matrix and hyper-edges are the row elements. If node *v* belongs to the hyper-edge *e*, *H*(*v, e*) = 1, and if it does not, *H*(*v, e*) = 0.

Based on H, the node degree of each vertex *v* is represented as

(2) $$\begin{array}{l} d\left(v\right)=\underset{e\in E}{\sum }H\left(v,e\right) \end{array}$$

The edge degree of hyper-edge *e* is represented as

(3) $$\begin{array}{l} \delta \left(e\right)=\underset{v\in V}{\sum }H\left(v,e\right) \end{array}$$

*D*_*v*_ and *D*_*e*_ denote the diagonal matrices of node degrees *d*(*v*) and hyper-edge degrees δ(*e*), respectively. The adjacency matrix A of the hyper-graph is defined as

(4) $$\begin{array}{l} A=HH^{T}-D_{v} \end{array}$$

*H*^*T*^ is the transpose of *H. A(i,j)* represents the number of hyper-edges containing the nodes *v*_*i*_ and *v*_*j*_.

### Hyper-network construction

According to the anatomical automatic labeling (Tzourio-Mazoyer et al., 2002) template, the brain can be parcellated into 90 anatomical regions of interest (ROIs; 45 in each hemisphere), with each ROI representing a node of the functional brain network. Each regional mean time series was regressed against the average cerebral spinal fluid and white matter signals as well as the six parameters from motion correction. According to the sparse linear regression method, the residuals were used to construct the hyper-network (Jie et al., 2016). Using the sparse linear regression model, a region can be represented as a linear combination of other regions, and its interactions with a few other regions can be obtained. Insignificant and false interactions are forced to zero.

The sparse linear regression model is represented as follows:

(5) $$\begin{array}{l} x_{m}=A_{m}\alpha_{m}+\tau_{m} \end{array}$$

*x*_*m*_ denotes the average time series of the designated *m-th* ROI. *A*_*m*_ = [*x*_1_, …, *x*_*m*−1_, 0, *x*_*m*+1_, …, *x*_*M*_] denotes the data matrix that includes the mean time series of the ROIs, except for the *m-th* ROI, which is set to 0. α_*m*_ denotes the weight vector of the degree of influence on other ROIs to the m-th ROI, and τ_*m*_ denotes a noise term. The corresponding ROIs of the nonzero element in α_*m*_ are the ROIs that interact with the designated brain region. The zero element indicates that the corresponding ROI is meaningless for accurately estimating the time series of the *m-th* ROI.

#### Solving the sparse linear regression model based on the lasso method

In the literature, the brain functional hyper-network is constructed using a sparse linear regression model, which is commonly solved by the lasso method. The optimization objective function is

(6) $$\begin{array}{l} \underset{\alpha_{m}}{\min}{\left||x_{m}-A_{m}\alpha_{m}|\right|}_{2}+\lambda {\left||\alpha_{m}|\right|}_{1} \end{array}$$

This solves the l_1_ norm, and *x*_*m*_, *A*_*m*_, and α_*m*_ have the same meaning as in Equation (5). || . ||_2_ denotes the l_2_ norm, and || . ||_1_ denotes the l_1_ norm. λ is the regularization parameter that controls the sparsity of the model, and different λ values correspond to different sparse solutions. The larger the value of λ, the sparser the model; that is, there are more zeros in the α_*m*_. The smaller the value of λ, the more dense the model; that is, there are more non-zeros in the α_*m*_. Therefore, the value of λ had a range. However, different experimental data will have different λ ranges and previous research standardized the range of λ from 0 to 1 based on λ_min_ and λ_max_ that made λ comparable (Liu et al., 2011). Thus, in current studies, the value of λ was set from 0.1 to 0.9 in increment of 0.1. The SLEP package (Liu et al., 2011) was adopted to solve the optimization problem. For each subject, a hyper-network was constructed by using sparse representation based on lasso method. A node was a brain region and a hyper-edge *e*_*m*_ included a centroid ROI (i.e., *m*-th ROI) and a few of other brain regions with the corresponding non-zero elements in the weight vector α_m_ computed in Formula (6). As mentioned above, a centroid ROI should generate a corresponding weight vector α_*m*_, which generated a corresponding hyper-edge. But to reflect the multi-level interactions among brain regions, for each ROI, a group of hyper-edges was generated by varying the value of λ in a range from 0.1 to 0.9. Thereby, for each subject, a 90 ^*^ 810 matrix was generated as a subject included 90 ROIs, which represented a hyper-network is constructed by lasso method.

#### Solving the sparse linear regression model based on the elastic net method

Although the lasso method has been successfully applied in many studies, it also has limitations. The lasso is not robust when the variables are high correlations and will select one randomly and neglect the others (Friedman et al., 2010b). Obviously, the lasso method cannot solve the grouping effect, and could cause some relevant brain areas to be missed in the process of selecting related areas within a designated region. If there is a group of brain regions among which the pairwise correlations are very high, then the lasso tends to select only one region from the group and does not care which one is selected (Zou and Trevor, 2005). The ideal hyper-edge construction method should be able to select the interacting brain regions as accurately as possible. To solve the problem of the grouping effect among brain regions, we propose two methods to improve the construction of a hyper-network: the elastic net method and the group lasso method.

The elastic net is an extension of the lasso that is robust to extreme correlations among the predictors (Friedman et al., 2010b). Similar to the lasso, the elastic net can also solve the problem of sparse representation. The difference is that the elastic net can overcome the limitation of the lasso to select related variables in a group when solving the linear regression model to construct the hyper-edges, thus addressing the grouping effect. The elastic net uses the mixed penalty term of l_1_ norm(lasso) and l_2_ norm(ridge regression), which can be represented as the following regularized objective function optimization problem:

(7) $$\begin{array}{l} \underset{\alpha_{m}}{\min}{\left||x_{m}-A_{m}\alpha_{m}|\right|}_{2}+\lambda_{1}{\left||\alpha_{m}|\right|}_{1}+\lambda_{2}||\alpha_{m}|{|}_{2}^{2} \end{array}$$

*x*_*m*_, *A*_*m*_, and α_*m*_ have the same meaning as in Equation (5). λ_1_ is the l_1_-norm regularization parameter, and λ_2_ is the regularization parameter for the squared l_2_-norm. Despite elastic net has one more parameter (l_2_-norm) than lasso, it greatly effects the calculation result and works well in solving the grouping effect (Furqan and Siyal, 2016). Because l_2_-norm (Hoerl and Kennard, 2000) performs well with many variables that are highly correlated and can effectively adjust the high correlation between independent variables so that the model can automatically choose related features in a group with grouping effect (Friedman et al., 2010b). Therefore, with l_1_ being for automatic variable selection and l_2_ encouraging grouped selection (Ogutu et al., 2012), the integration of l_1_ and l_2_ should greatly improve the construction of hyper-network. Similar to the lasso, we constructed a hyper-network for each subject, in which ROIs were the nodes and the hyper-edge *e*_*m*_ included a centroid ROI (i.e., *m*-th ROI) and the corresponding ROIs of the nonzero elements in α_*m*_ computed in formula (7). Like the lasso method, a centroid ROI corresponded to a hyper-edge. For each ROI, with the value of λ_2_ fixed, a group of hyper-edges was generated by varying the value of λ_1_ in a range from 0.1 to 0.9 in increments of 0.1. In the experiment, the value of λ_2_ was chosen as 0.2 because this was found to provide the highest classification accuracy (see the Methodology section for a detailed description of the analysis).

#### Solving the sparse linear regression model based on the group lasso method

The clustering method was used to group the strongly correlated brain regions, then the group lasso method was adopted to construct the hyper-edges, which can also help to solve the grouping effect among brain regions. The lasso and elastic net methods are used to select single variables (Yuan and Lin, 2006), whereas the group lasso can select groups of variables based on predefined variable groups (Meier et al., 2008). We first clustered the 90 brain regions according to the average time series of ROIs when constructing the hyper-network. Here, the k-medoids (Park and Jun, 2009) algorithm was adopted. First, the pairwise similarity values among brain regions were calculated: higher values indicate greater similarity between the two samples. The clustering classified the brain areas into *k* groups, each of which represented a class of objects, under two conditions: (1) each group must contain at least one object and (2) each object must belong to a group. To stabilize the clustering as much as possible, *k*-means [Arthur and Vassilvitskii, 2007] was used to select the *k* initial cluster centers. A point was randomly selected as the first initial cluster center, and each subsequent center was chosen randomly from the remaining data points with a probability proportional to its distance from the point closest to the existing cluster center. We repeated the clustering 10 times to select the best clustering result. The *k*-value used in the experiment can affect the network structure and classification performance. The highest classification accuracy was obtained when *k* was equal to 48 (see the Methodology section). Then, the group lasso was adopted to select the brain areas for the construction of the hyper-edge, with the following optimization objective function:

(8) $$\begin{array}{l} \underset{\alpha_{m}}{\min}{\left||x_{m}-A_{m}\alpha_{m}|\right|}_{2}+\beta \overset{k}{\underset{i=1}{\sum }}{\left||\alpha_{m}G_{i}|\right|}_{2,1} \end{array}$$

β is the l_2,1_-norm regularization parameter, which is regarded as an intermediate between the l_1_- norm and l_2_-norm penalty, with different values corresponding to different degrees of sparsity. The greater the value of β, the sparser the model, and the fewer the number of groups selected. It can perform variable selection effectively in the group level (Yuan and Lin, 2006). That is, if there is a group of brain regions in which the pairwise correlations are relatively high, the group lasso considers this group as a whole and determines whether it is important to the problem. The α_*m*_ is divided into k non-overlapping groups by clustering, and α_*m*_*G*_*i*_ represents the *i-th* group. Similarly, we constructed the hyper-network with ROIs as the nodes, the hyper-edge including the *m-th* ROI and the corresponding ROIs of the nonzero elements in α_*m*_ computed in Formula (8). A hyper-edge was generated by a centroid ROI. For each ROI, a group of hyper-edges was generated by varying the value of β in a range from 0.1 to 0.9 in increments of 0.1.

### Feature extraction

The feature extraction was carried out on the three hyper-networks constructed by the three methods. The clustering coefficient is widely applied as a metric to measure the local characteristics of a network. However, the clustering coefficient in a hyper-network is not always defined in exactly the same way. Here, the feature extraction selected three different definitions of the clustering coefficient (Gallagher and Goldberg, 2013), reflecting different angles. The first type of clustering coefficient, *HCC*^1^, computes the number of adjacent nodes that have connections not facilitated by node *v*. The second type, *HCC*^2^, calculates the number of adjacent nodes that have connections facilitated by node *v*. The third type, *HCC*^3^, calculates the amount of overlap amongst adjacent hyper-edges of node *v*. The formula is as follows:

(9) $$\begin{array}{l} HCC^{1}\left(v\right)=\frac{2\sum_{u,t\in N\left(v\right)}I\left(u,t,\neg v\right)}{|N\left(v\right)|\left(\left|N\left(v\right)-1\right|\right)} \end{array}$$

*HCC*^1^(*v*) represents the first type of clustering coefficient, and *u*,*t*,*v* represent the nodes.*N*(*v*) = {*u* ∈ *V* : ∃*e* ∈ *E, u, v* ∈ *e*}, where *V* represents a set of nodes, *E* represents a set of hyper-edges, *e* represents a hyper-edge, and *N*(*v*) represents the set of nodes of other hyper-edges containing node *v*. If ∃*e*_*i*_ ∈ *E*, such as *u, t* ∈ *e*_*i*_, but *v* ∉ *e*_*i*_, then *I*(*u, t*, ¬*v*) = 1; otherwise, *I*(*u, t*, ¬*v*) = 0.

(10) $$\begin{array}{l} HCC^{2}\left(v\right)=\frac{2\sum_{u,t\in N\left(v\right)}I^{\prime }\left(u,t,v\right)}{|N\left(v\right)|\left(|N\left(v\right)|-1\right)} \end{array}$$

*HCC*^2^(*v*) represents the second type of clustering coefficient. *u*,*t*,*v* and *N*(*v*) have the same meaning as in Equation (9). If ∃*e*_*i*_ ∈ *E*, such as *u, t, v* ∈ *e*_*i*_, then *I*′(*u, t, v*) = 1; otherwise, *I*′(*u, t, v*) = 0.

(11) $$\begin{array}{l} HCC^{3}\left(v\right)=\frac{2\sum_{e\in S\left(v\right)}\left(|e|-1\right)-|N\left(v\right)|}{|N\left(v\right)|\left(|S\left(v\right)|-1\right)} \end{array}$$

*HCC*^3^(*v*) represents the third type of clustering coefficient. |*e*| represents the number of nodes in a hyper-edge. *v* and *N*(*v*) have the same meaning as in Equation (9). *S*(*v*) = {*e*_*i*_ ∈ *E* : *v* ∈ *e*_*i*_}, where *v* represents a node, *e*_*i*_ represents a hyper-edge, and *S*(*v*) represents the set of hyper-edges containing node *v*.

The three clustering coefficients reflect the local clustering properties of the hyper-network from different perspectives. The definition of each clustering coefficient was extracted from the hyper-network as features. Multiple linear regression analyses were applied to evaluate the confounding effects of age, gender, and educational attainment for each network property. Because the three clustering coefficients define local properties, for simplicity, we calculated the average clustering coefficient (average HCC^1^, HCC^2^, and HCC^3^) for each subject (using the average metric values for 90 brain areas), and added it to the multiple linear regression as the independent variable. The results showed that no significant correlations were found between the network properties and the potential confounding variables. (The results are presented in detail in Supplemental Text S2).

### Feature selection and classification

Some of the features extracted from the hyper-network may be irrelevant or redundant. To select the key features for classification, the most discriminative features were chosen according to the statistical difference analysis. Specifically, Kolmogorov and Smirnov's nonparametric permutation test (Fasano and Franceschini, 1987) was performed to compare 270 node properties between the MDD group and the control group, corrected using Benjamini and Hochberg's false-discovery rate (FDR) method (*q* = 0.05) (Benjamini and Hochberg, 1995). The local properties with significant between-group differences according to the nonparametric permutation test were used as the classification features to construct the classification model.

Classifier training was performed using the libsvm classification package (http://www.csie.ntu.edu.tw/~cjlin/libsvm/). The radial basis function (RBF) kernel was adopted for the classification, and leave-one-out cross-validation was used to evaluate the performance. Suppose there are *N* features, with each feature taking a turn as the testing set, and the remaining *N*−1 features as the training set. The parameter optimizations of c and g were carried out by the K-fold cross-validation method using the training set (Hsu et al., 2003). The optimal parameters of c and g which can achieve the highest training validation classification accuracy were chosen to establish *N* different models. The classification test was performed and the average classification accuracy of the *N* models was selected. The mean and standard deviation of the classification features needed to be calculated for standardization. Because the random selection of the initial seed point in the group lasso method affects the classification result, the mean of 50 experiments was calculated as the final classification result. In addition, the Relief algorithm (Kira and Rendell, 1992) was selected to compare the importance of the chosen features (i.e., the extent of their contribution to the classification) of the three methods in section Classification Performance.

## Experiments and results

### Comparison of network structure based on three methods

We performed the following analyses to determine whether there were significant differences among the hyper-networks constructed by the three methods.

**Figure 3:**
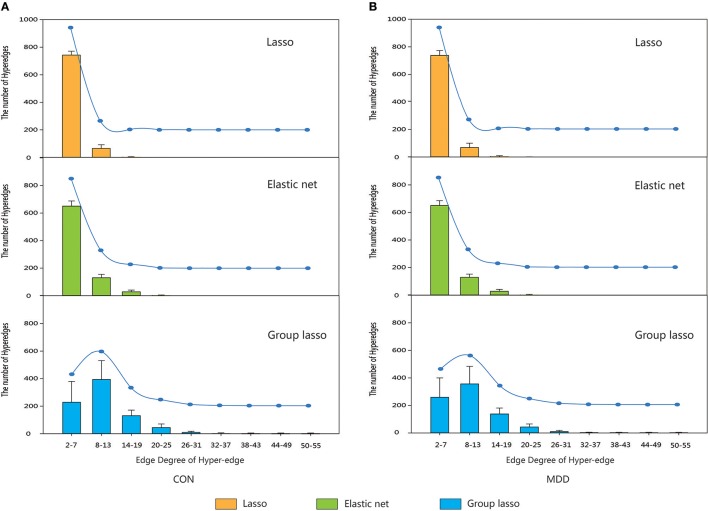
The distribution of the edge degree of hyper-edge obtained by three methods. Orange indicate the distribution of the edge degree of hyper-edge based on lasso method. Green indicate the distribution of the edge degree of hyper-edge based on elastic net method. Blue indicate the distribution of the edge degree of hyper-edge based on group lasso method. Error bars show standard deviation. **(A)** CON group. **(B)** MDD group. CON, normal control; MDD, major depressive disorder.

Hyper-networks were constructed for subjects in the control and MDD groups and the hyper-edges were analyzed. The edge degrees of the hyper-edges constructed by the three methods were computed and their distribution is shown in Figure 3.

The results indicated that for both the MDD and the control group, the edge degrees of hyper-edges constructed by lasso and elastic net methods most lied in Equations (2)–(7), and the distributions were also close. The hyper-edges constructed by the group lasso method were different, with a broader range of edge degrees than the other two methods and a relatively discrete distribution.

**Figure 4:**
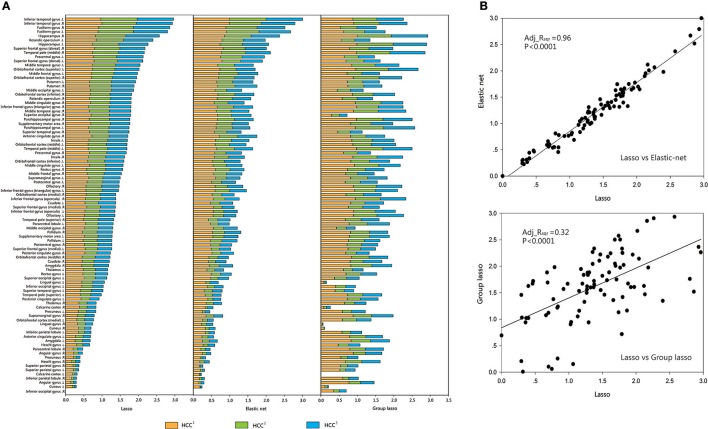
Comparison and correlation analysis about the standardized metric values in CON Group. **(A)** Comparison that the brain regions were sorted according to the size of the standardized metric value in the lasso method, and according to the corresponding sequence about the other two methods for comparison in CON group. **(B)** Correlation analysis. Correlation analysis was performed to verify the associations between the network metrics obtained by the two proposed methods and the original method. HCC^1^ indicate the first type of clustering coefficient; HCC^2^ indicate the second type of clustering coefficient; HCC^3^ indicate the third type of clustering coefficient. *P* indicate the significance of correlation analysis. Adj.R_sqr_, adjusted R square.

**Figure 5:**
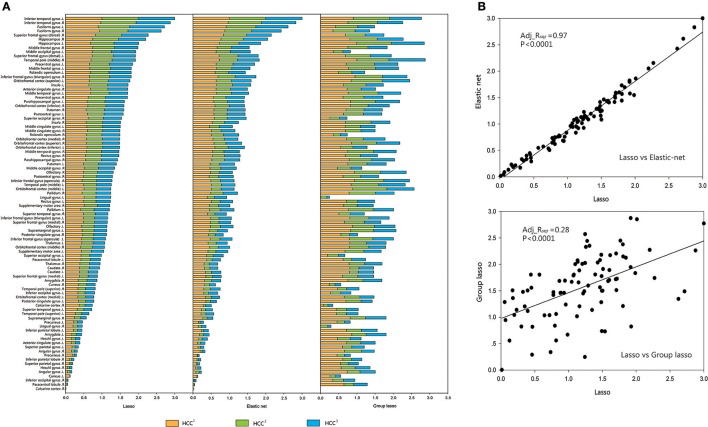
Comparison and correlation analysis about the standardized metric values in MDD Group. **(A)** Comparison that the brain regions were sorted according to the size of the standardized metric value in the lasso method, and according to the corresponding sequence about the other two methods for comparison in MDD group. **(B)** Correlation analysis. Correlation analysis was performed to verify the associations between the network metrics obtained by the two proposed methods and the original method. HCC^1^ HCC^1^ indicate the first type of clustering coefficient; HCC^2^ indicate the second type of clustering coefficient; HCC^3^ indicate the third type of clustering coefficient. *P* indicate the significance of correlation analysis. Adj.R_sqr_, adjusted R square.

For each brain region, the mean value of each group (CON or MDD) was computed for each type of clustering coefficient, and the acquired data were standardized. The brain regions were sorted according to the size of the standardized metric value for the lasso method, and according to the sequence of comparison for the other two methods. A regression analysis was performed to verify the associations between the network metrics obtained by the two proposed methods and the original method. The results showed that the lasso method was strongly related to the elastic net method and weakly related to the group lasso method (Figures 4, 5).

**Figure 6:**
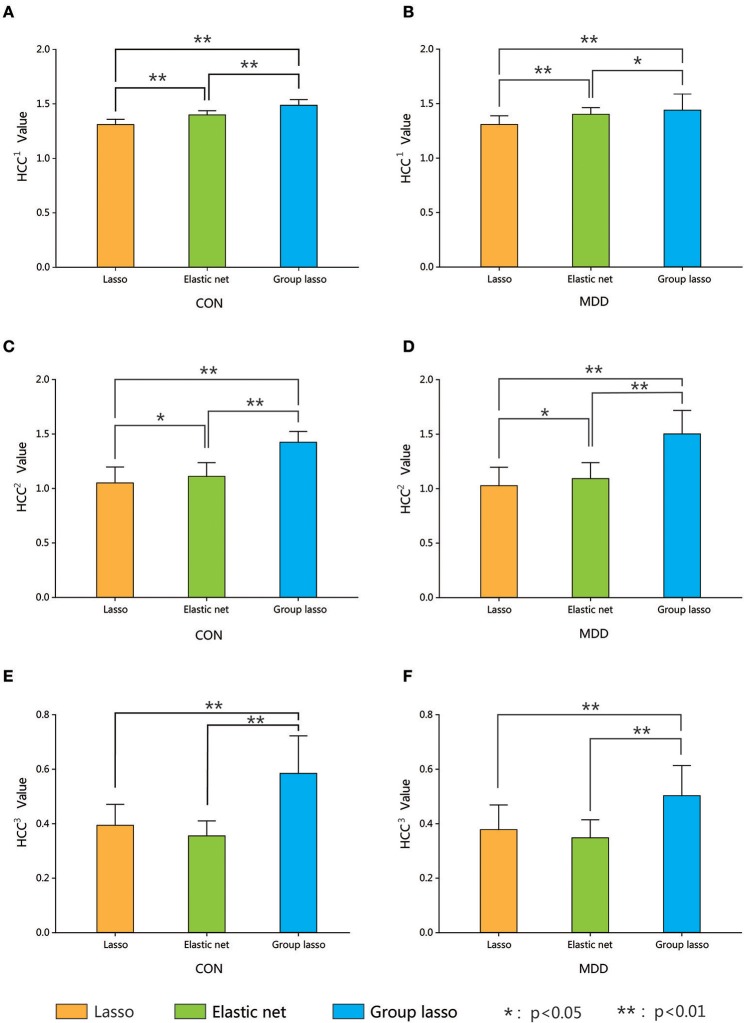
Comparison among three hyper-networks about three kinds of average clustering coefficients. Error bars show standard deviation. Asterisks indicate a significant difference. *p < 0.05, **p < 0.01. CON, normal control; MDD, major depressive disorder. HCC^1^ indicate the first type of clustering coefficient; HCC^2^ indicate the second type of clustering coefficient; HCC^3^ indicate the third type of clustering coefficient.

The average clustering coefficients (average HCC^1^, HCC^2^, and HCC^3^) of each subject (using the average metric values for 90 brain areas) were calculated. Nonparametric permutation tests were used to compare the differences in the average HCC^1^, HCC^2^, and HCC^3^ among the hyper-networks constructed by the three methods in the depression group and the normal control group, respectively, and the result was corrected using the FDR method. Figure 6 shows the average clustering coefficients of the hyper-networks for the two groups; the results suggest that the hyper-networks obtained by the three methods contained structural differences.

### Brain regions with significant differences

**Figure 7:**
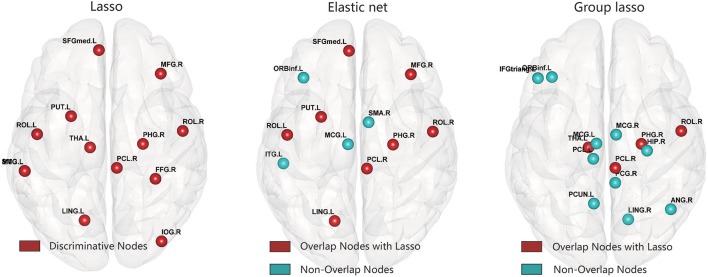
Abnormal brain regions were mapped onto the cortical surfaces using BrainNet viewer software.

After constructing the hyper-network and extracting the features based on the three methods, for each feature we carried out a nonparametric permutation test to evaluate the difference between the MDD and control groups for all subjects, corrected using the FDR method. Table 2 and Figure 7 illustrate the brain regions that showed significant between-group differences in the three hyper-network construction methods.

**Table 2:** Abnormal brain regions and significance.

**ROIs**	***P*****-value**		
	**Clustering coefficient**	**Clustering coefficient**	**Clustering coefficient**
**LASSO**			
Left supramarginal gyrus	**0.048**	0.214	0.118
Left rolandic operculum	0.118	0.118	**0.007**
Right rolandic operculum	0.303	0.094	**0.045**
Left superior frontal gyrus, medial	0.207	0.055	**0.007**
Right parahippocampal gyrus	0.638	**0.015**	**0.005**
Left thalamus	0.294	**0.049**	0.252
Left putamen	0.214	0.122	**0.047**
Right middle frontal gyrus	**0.019**	0.157	0.169
Left lingual gyrus	**0.017**	0.260	0.109
Right inferior occipital gyrus	0.060	**0.039**	**0.045**
Right fusiform gyrus	0.792	**0.047**	0.612
Right Paracentral lobule	0.393	**0.049**	0.090
Left middle temporal gyrus	0.804	**0.037**	0.181
**ELASTIC NET**			
Right middle frontal gyrus	**0.009**	**0.066**	0.573
Left inferior frontal gyrus, orbital part	**0.043**	**0.043**	0.303
Left rolandic operculum	**0.040**	0.087	**0.038**
Right rolandic operculum	**0.045**	**0.045**	0.053
Right supplementary motor area	**0.024**	0.091	**0.049**
Left superior frontal gyrus, medial	0.332	0.286	**0.047**
Left median cingulate and paracingulate gyri	0.850	**0.040**	**0.021**
Right parahippocampal gyrus	0.313	**0.019**	0.080
Left lingual gyrus	0.090	0.098	**0.008**
Right Paracentral lobule	0.612	0.229	**0.017**
Left putamen	**0.047**	**0.011**	**0.041**
Left inferior temporal gyrus	0.665	**0.008**	0.994
**GROUP LASSO**			
Left inferior frontal gyrus, triangular part	**0.007**	0.968	0.063
Left inferior frontal gyrus, orbital part	**0.017**	0.817	**0.007**
Right rolandic operculum	0.265	0.991	**0.003**
Left median cingulate and paracingulate gyri	**0.038**	0.461	**0.005**
Right median cingulate and paracingulate gyri	**0.012**	0.201	**0.001**
Right posterior cingulate gyrus	0.303	0.341	**0.001**
Right hippocampus	**0.001**	0.058	**0.017**
Right parahippocampal gyrus	**0.006**	0.586	**0.016**
Right lingual gyrus	0.351	**0.006**	0.665
Right angular gyrus	**0.004**	**0.045**	0.080
Left precuneus	0.252	0.322	**0.005**
left paracentral lobule	0.094	0.147	**0.009**
Right paracentral lobule	0.252	0.586	**0.002**
Left thalamus	0.087	**0.002**	0.404

*Bold indicates significance p < 0.05*.

Comparison of the regions obtained by the elastic net and lasso methods indicated that there were more overlapping than non-overlapping regions, including partial frontal areas [left superior frontal gyrus (medial), right middle frontal gyrus, right paracentral lobule], the bilateral rolandic operculums, partial limbic lobe (right parahippocampal gyrus), partial subcortical gray nucleus (left putamen), and partial occipital area (left lingual gyrus). The hyper-networks produced by the lasso and elastic net methods were similar, so there were more overlapping than non-overlapping regions between the lasso and elastic net methods. The group lasso result was much different from that of the lasso method; there were more non-overlapping than overlapping regions, including the left inferior frontal gyrus (triangular part), left inferior frontal gyrus (orbital part), left paracentral lobule, left median cingulate and paracingulate gyri, right median cingulate and paracingulate gyri, right posterior cingulate gyrus, right hippocampus, right angular gyrus, left precuneus, and right lingual gyrus. These brain regions are mainly concentrated in the frontal lobe and limbic system.

### Classification performance

We assessed the classification performance by measuring the accuracy (the proportion of subjects correctly identified), the sensitivity (the proportion of patients correctly identified), the specificity (the proportion of controls correctly identified), and balanced accuracy. Balanced accuracy is defined as the arithmetic mean of sensitivity and specificity, and can avoid inflated performance on imbalanced datasets (Velez et al., 2007).

We evaluated the classification performance of the three brain network classification methods based on the lasso, elastic net, and group lasso, and compared them with the traditional network classification method (denoted as TCN). The traditional network, based on Pearson's correlations, was constructed with sparsity set from 5 to 40%. Three local properties were calculated: degree, centrality degree, and node efficiency. To characterize the overall characteristics of the metrics in the selected threshold space, the AUC value of each property was calculated. We selected the AUC values of the local properties that showed a significant between-group difference in the KS nonparametric permutation test as the classification features. The classification results of the comparison methods are summarized in Table 3.

**Table 3:** The classification performance of the four methods.

**Methods**	**Accuracy**	**Sencitivity (%)**	**Specificity (%)**	**BAC (%)**
TCN	71	79	64	71.5
Lasso	83.33	84.21	82.14	83.175
Group lasso	80.30	84.21	75	79.60
Elastic net	86.36	92.10	81.57	86.83

*TCN, traditional connectivity network method; lasso, hyper-network method based on lasso; elastic net, hyper-network method based on elastic net; group lasso, hyper-network method based on group lasso; BAC, balanced accuracy*.

**Figure 8:**
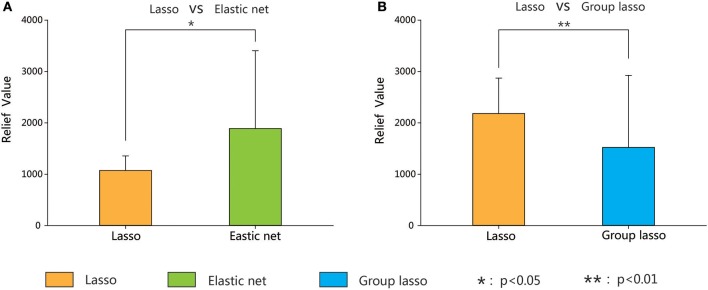
Classification weight of different discriminative regions between lasso method and the other two methods (elastic net and group lasso methods) corresponding features. Error bars show standard deviation. Asterisks indicate a significant difference. *p < 0.05, **p < 0.01.

To compare the importance of the chosen features (i.e., the extent of their contribution to the classification) of the three methods, the brain areas with significant between-group differences based on the lasso method were compared with the results obtained by the other two methods. We combined the brain areas with no overlap between the two methods (the lasso and the elastic net, and the lasso and the group lasso). The Relief algorithm (Kira and Rendell, 1992) was adopted to calculate the weights of the corresponding features of these brain areas. The results in Figure 8 indicate that the classification weight of the elastic net method was higher than that of the lasso method and that of the group lasso was lower.

**Figure 9:**
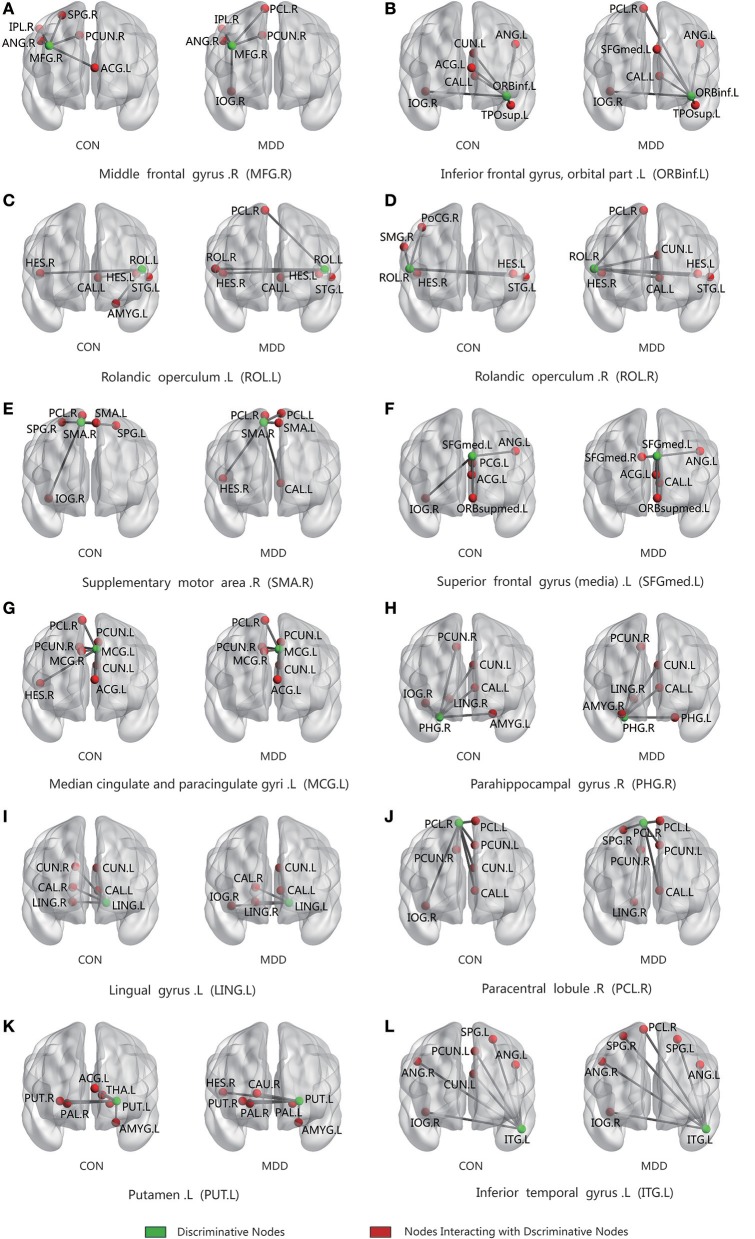
Connected patterns of abnormal brain regions in hyper-network constructed by elastic net method; The average hyper-edges for CON(left) and MDD(right) groups based on 12 ROIs (the green node) listed in Table 2 (elastic net) with λ_1_ = 0.3. Here, each sub-figure denotes a hyper-edge constructed by the corresponding ROI, and the green nodes in each subgraph represent the brain areas with significant differences between-group. L, left; R, right; CON, normal control. MDD, major depressive disorder.

For all four methods, the best classification accuracy was obtained using the hyper-network construction method based on elastic net, so we analyzed the differences in the connection patterns between the MDD and control groups with the elastic net method. The average hyper-edges of the MDD and control groups were calculated based on the brain areas with significant between-group differences in the elastic net method. First, for each group of subjects, we constructed the hyper-edges according to the fixed value of λ_1_ and Equation (7), computing the number of occurrences of each brain area in each group of hyper-edges. Then, the average of edge degrees of the hyper-edges for all subjects was calculated, denoted as d. If it was not an integer, then d was rounded to the nearest integer (greater than or equal to). Finally, the number of occurrences of the brain area in each group of hyper-edges was sorted from high to low, and the first d brain areas were chosen to construct the corresponding average hyper-edges. Figure 9 illustrates the average hyper-edges based on the 12 ROIs listed in Table 2 (elastic net) with λ_1_ = 0.3. The left side of each subgraph in Figure 9 shows the average hyper-edges of the control group and the right side shows those of the depression group. The green nodes in each subgraph represent the brain areas with significant between-group differences.

Figure 9 shows that the hyper-edges of the MDD and control groups were discrepant. For example, Figure 9D shows that in the MDD group, the right rolandic operculum (ROL.R) mainly interacted with the right paracentral lobule (PCL.R), left cuneus (CUN.L), left heschl gyrus (HES.L), right heschl gyrus (HES.R), left calcarine fissure and surrounding cortex (CAL.L), and the left superior temporal gyrus (STG.L); however, in the control group, the right rolandic operculum (ROL.R) mainly interacted with the right postcentral gyrus (POCG.R), right supramarginal gyrus (SMG.R), left superior temporal gyrus (STG.L), left heschl gyrus (HES.L), and right heschl gyrus (HES.R).

## Discussion

The accuracy of brain network classification based on a hyper-network is strongly dependent on the network construction. A new method of hyper-network construction was recently proposed; however, the method was unable to obtain some relevant brain regions when establishing the hyper-edges because of the grouping effect among regions. To solve this problem, we proposed two new methods for constructing a hyper-network based on the elastic net and the group lasso. In the lasso method proposed by Jie et al. (2016), the optimal objective function for solving the sparse linear regression model includes the loss function and the l_1_ norm penalty term. The penalty term makes it possible to continuously compress and select variables automatically and simultaneously. The optimal objective function of elastic net method adds an l_2_ norm penalty term to the lasso. Recent studies (Zou and Trevor, 2005; De Mol et al., 2008) have shown that the l_2_ norm can effectively adjust the high correlations among the independent variables so that the model can automatically select the relevant variables in a group with a grouping effect. The group lasso method introduces the penalty function for the variable grouping factor to select variables using the l_2,1_ norm, based on predefined variable groups.

There were differences among the hyper-networks constructed by the three methods. The MDD and control groups had similar distributions according to the analysis of the hyper-edge degrees. The lasso and elastic net methods produced very similar results for the range of edge degrees and the number of hyper-edges within each range. However, the results for the group lasso method were quite different. The range of edge degrees was 2–55, indicating the looseness of the constraint, and there were some hyper-edges with more nodes. We conclude that the structure of the hyper-network obtained by the lasso was similar to that obtained by the elastic net, but very different from that obtained by the group lasso. This conclusion was confirmed by the comparison of metrics. When constructing the hyper-edge using the lasso method, only one brain region was selected from a group because of the grouping effect. The elastic net method helped to select the related brain regions by adding the l_2_ norm, which can select some brain regions from that group. When the group lasso method selected a brain region from the group, all of the brain regions in the group were considered relevant. Therefore, the lasso method was the strictest, the group lasso method was the loosest, and the elastic net method was moderate.

We obtained a similar conclusion from the correlation analysis of the metrics. The metrics of all subjects in each group were averaged across brain regions, which were sorted after standardization. Linear regression analyses were performed on the metrics obtained by the lasso and the other two methods. The standardized metric values based on brain regions showed significant associations between the elastic net and the lasso methods (CON group: adj_R_sqr_ = 0.96, MDD group: adj_R_sqr_ = 0.97), but significant differences between the group lasso and the lasso methods (CON group: Adj_R_sqr_ = 0.32, MDD group: Adj_R_sqr_ = 0.28).

Furthermore, analysis of the average clustering coefficients (average *HCC*^1^, *HCC*^2^, and *HCC*^3^) showed significant differences in the average *HCC*^1^ and average *HCC*^2^ among the three hyper-networks for both the MDD and control groups. The average *HCC*^3^ showed significant differences between the group lasso and the other two methods. No significant difference (*p* > 0.05 FDR corrected, *q* = 0.05) was found between the lasso and elastic net methods. These results indicated that there were structural differences among the hyper-networks constructed by the three methods. The lasso and elastic net produced similar hyper-network structures, but the structure produced by the lasso was very different from that produced by the group lasso. In terms of network construction constraints, the lasso was the strictest, the group lasso was the loosest, and the elastic net method was in-between. We believe that these results are attributable to differences in the different methods' ability to resolve the grouping effect.

The areas with statistically significant between-group differences were not identical among the three methods. The lasso and elastic net methods had more overlapping than non-overlapping regions, while the group lasso and lasso methods showed the opposite result. This result also verified the conclusion we obtained from the other analyses.

The best classification accuracy was obtained based on the elastic net method, so we used the hyper-network based on this method to analyze the abnormal brain regions. Statistical analysis of this hyper-network identified 12 abnormal regions: some partial frontal areas (right middle frontal gyrus, left inferior frontal gyrus (orbital part), right supplementary motor area, left superior frontal gyrus (medial), right paracentral lobule), bilateral rolandic operculums, partial limbic lobes (left median cingulate and paracingulate gyri, right parahippocampal gyrus), partial subcortical gray nucleus (left putamen), partial temporal lobe (left inferior temporal gyrus), and partial occipital area (left lingual gyrus). These regions are consistent with previous results reported in the literature (Table 4). The value of brain network research is in identifying changes in the brain network. Analysis of the connection patterns among the brain regions with between-group differences identified by the elastic net method and the other regions revealed different interaction patterns between the MDD and control groups.

**Table 4:** ROIs selected from the other literature about depression.

**ROI**	**References**
Right middle frontal gyrus	Guo et al., 2012
Left inferior frontal gyrus, orbital part	Jin et al., 2011; Guo et al., 2012; Lord et al., 2012
Rolandic operculum	Zhu et al., 2016
Right supplementary motor area	Liu et al., 2012
Left superior frontal gyrus, medial	Jin et al., 2011
Left median cingulate and paracingulate gyri	Guo et al., 2012; Zhu et al., 2016
Right parahippocampal gyrus	Qiu et al., 2014
Left lingual gyrus	Lord et al., 2012; Qiu et al., 2014
Right paracentral lobule	Qiu et al., 2014
Left putamen	Lord et al., 2012; Gong et al., 2014
Left inferior temporal gyrus	Gong et al., 2014

Three methods of hyper-network construction and a correlation method were used to classify 38 patients with depression and 28 control subjects. The results suggest that hyper-network methods can improve brain network classification performance. The classification accuracy of our two proposed methods exceeded 80%. The elastic net hyper-network construction method outperformed the others, and the accuracy reached 86.36% when the value of λ_2_ was set to 0.2. The classification result obtained by the group lasso method was not as good as the original method. The underlying reasons are that the k-medoids clustering method introduces uncertain parameters, and it easily falls into a local optimum. Although we used the idea of k-means++ to optimize the selection of the initial points, the random selection of the first initial cluster center still led to unstable results. Moreover, the group lasso cannot choose variables flexibly within a group.

Based on the lasso and elastic net methods, we obtained two groups of brain regions with between-group differences, and then calculated the classification weight of the corresponding features of the non-overlapping brain regions between two group regions. The Relief algorithm is used to weight features according to the correlation between a feature and a category. Features with stronger weights have better classification ability (Kira and Rendell, 1992). The weights of the features obtained by the elastic net method were significantly larger than those obtained by the lasso method, which in turn were significantly greater than those obtained by the group lasso method. The results imply that a moderate connection constraint (elastic net) can acquire classification features more effectively than a constraint that is too strict (lasso) or too loose (group lasso).

## Methodology

The construction of hyper-network is based on sparse regression model with penalties. By using linear sparse regression model, a brain region can be characterized by a linear combination of a few other brain regions, which may be obtained with pairwise correlation from the view of math method. However, by introducing the penalties control, one brain region can be interacted with a few other brain regions while forcing insignificant interactions to zero, and regard these brain regions as a hyper-edge to construct the hyper-network, which was a multivariate expression that described the multiple interactions of brain regions to interpret the hyper-network topology. For more precisely diagnosis of brain diseases, we proposed elastic net and group lasso sparse regression methods to construct the hyper-network taking into the grouping effect.

The performances of the proposed classification methods depend on the selection of certain parameters, such as the cluster number *k*, the hyper-network construction model parameters λ_1_ and λ_2_, the SVM model parameters *c* and *g*. We compared brain network classification methods based on the group lasso and elastic net methods to explore this problem.

### Effect of cluster number k

**Figure 10:**
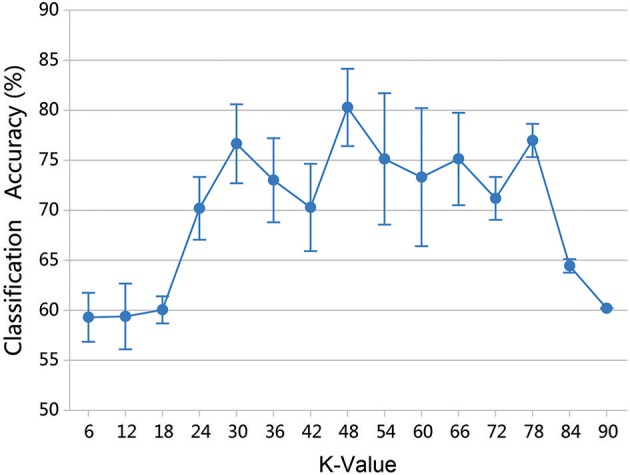
Classification accuracy of different *k* values. The results were obtained by 50 experiments at each *k*-value. Error bars show standard deviation.

Parameter *k* is the number of clusters in the group lasso method, and varying the value of *k* produces different network structures and classification results. To assess the effect on classification performance, the range of *k* was set at (6, 90) with a step size of 6. Because the random selection of the first initial seed point can affect the results, 50 experiments were performed for each *k* value, and the mean accuracy was chosen as the final classification result. Figure 10 shows that the highest accuracy of 80.30% was obtained when *k* equaled 48.

### Effect of regularization parameters λ_1_ and λ_2_

It has been shown in previous studies that the parameter λ has a significant impact to hyper-network topology. The parameter λ determined the sparsity and scale of the network. When λ was too small, the network would be too coarse and involve much noise. When λ was too large, the network would be too sparse (Lv et al., 2015). Moreover, the reliability of the network topology, especially the modularity, was sensitive to the sparsity controlled by the parameter λ (Li and Wang, 2015). Besides, the parameter λ had an effect on the classification performance. The classification model parameter, especially λ, was sensitive to classification accuracy. Since the selection of λ was not specified formulation, the proper choice of the parameter λ is very important for constructing hyper-networks and for classification. There were also some methods to choose λ to optimize the network topology reliability and classification performance in previous studies (Braun et al., 2012; Li and Wang, 2015; Qiao et al., 2016). However, there are the same limitations that it had low reliable network topology. This research showed that only when λ is 0.01 (indicating that all the nodes on the network are on a hyper-edge), the network had a high reliability (Li and Wang, 2015). Thus, multi-level λ setting method was proposed (Jie et al., 2016). Different from single λ setting, multi-level λ can combine several λ to provide more network topology information. And the multi-level λ setting can avoid random selection leading to single λ setting and reduce the impact of low reliability caused by a single network topology. In current studies, λ_1_ is the regularization parameter of the l_1_ norm, which is biased to control the model sparsity. Multi-level λ_1_ setting was introduced and the interval was set 0.1. λ_2_ was the regularization parameter of the l_2_ norm, which encouraged the influence of grouping. As different values of λ_2_ had different grouping effects and lead different classification performance, the interval was set 0.1. Different values of λ_1_ and λ_2_ produce different solutions.

**Figure 11:**
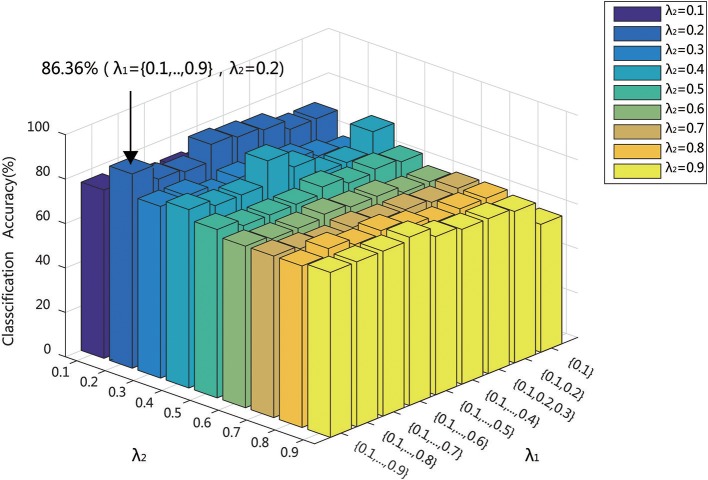
Classification accuracy of different network construction parameters (λ_1_,λ_2_).

In the multi-level λ setting, it is important that how to get the optimizing combination of λ. If Enumeration method was adopted, the computation consumption was too huge. Thus, in current studies, λ_1_ used a series of ascending order combination, which is {0.1}, {0.1, 0.2}, {0.1, 0.2, 0.3}, ……, {0.1, 0.2, ……, 0.9}, while λ_2_ are adopted, which is 0.1, 0.2,…,0.9. For λ_2_ value, nine sets of λ_1_ were used to construct the hyper-edges, {0.1}, {0.1, 0.2}, {0.1, 0.2, 0.3}, ……, {0.1, 0.2, ……, 0.9}, which formed the hyper-network. Keep the small λ values in the combinations as more as possible, which indicated that more nodes are connected in the constructed hyper-edges. It was thought that the hyper-edges with many nodes could depict the underlying relationship among several brain regions. Then, the features were extracted for classification. The classification results in Figure 11 indicated that the highest accuracy of 86.36% was obtained when λ_2_ = 0.2 and λ_1_ = {0.1,0.2,…, 0.9}. When λ_1_ = {0.1}, the classification accuracies were <60% because when λ_1_ used only one value, some nodes were contained in only one hyper-edge. The denominator of *HCC*^3^ is then zero, which makes it impossible to create an effective model for classification.

### Effect of SVM classification parameters *c* and *g*

SVM classification is widely applied in various fields. It is the key to selecting the kernel function in the classification. The RBF kernel function was chosen in this experiment. The two parameters of the SVM model, the penalty factor *c* and the kernel parameter *g*, strongly influence the classification, and thus it is important to finding the optimal values. The penalty factor *c* is used to control the compromise between the model complexity and the approximation error. If *c* is too large, the data fitting and the complexity of the learning machine will be too high. There is an necessary process to avoid overfitting when designing the classifier. on the contrary, if *c* is too small, the punishment for the empirical error will be small, the learning complexity of the machine and the data fitting will be low. When the overfitting or underfitting occurs, the generalization ability of the classifier will be reduced to influence the classification performance. The value of *g* of the RBF kernel function is also important to directly affect the classification accuracy of the model.

**Figure 12:**
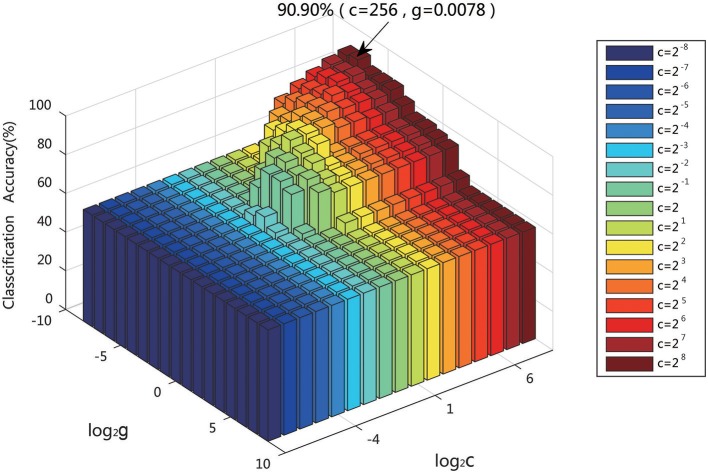
Training classification accuracy of different SVM parameters (*c, g*).

For given values of *c* and *g*, the K-fold cross-validation method was used to acquire the training set validation accuracy. The values of *c* and *g* that generated the highest validation classification accuracy were chosen as the optimum parameters. The parameters of *c* and *g* were set at [2^−8^, 2^8^], with a step of 1. Figure 12 displays the result when using the classification features as a training set to conduct the parameter optimization of *c, g*, which indicates that the highest training set validation accuracy of 90.90% was achieved when *c* = 256 and *g* = 0.0078.

### Limitations

The current study had three major limitations. First, the parameters of the hyper-network model in the experiment were a proportion of the corresponding parameters for the sparsest solution. The precise values were difficult to determine due to technical limitations. Furthermore, in the group lasso method, the random selection of the initial seed points and differences in the number of clusters *k* can change the network structure and the classification result. The construction of stable hyper-edges is expected to further improve the hyper-network. In addition, excessive analysis was not performed for comparing the current metrics (HCC1 2 3) and other clustering metrics in the experiment. The selection of better clustering metrics can be tried in follow-up research.

## Conclusion

The original method of hyper-network construction used the lasso to solve the sparse linear regression model. The method was limited because some related regions could not be chosen because of the grouping effect among brain regions in the process of establishing hyper-edges. To solve this problem, elastic net and group lasso methods were used to construct the hyper-networks. Analyses of the hyper-edges, brain area metrics, and average metrics implied that there were structural differences among the hyper-networks constructed by the three methods. The hyper-network obtained by the lasso was similar to that obtained by the elastic net but very different from that obtained by the group lasso. The lasso imposed a strict constraint on the network construction, the group lasso a loose constraint, and the elastic net a moderate constraint. Considering the potential reasons, we concluded that the existence of the grouping effect and differences in the methods' ability to resolve it led to these consequences. Different constraint conditions resulted in varying classification accuracies. The elastic net method outperformed the others, and the group lasso method was not as good as the original method. Meanwhile, the elastic net method had a higher classification weight than the lasso method, and the group lasso method had a lower classification weight. The results implied that a moderate connection constraint (elastic net) produced the most effective classification features, whereas stricter (lasso) and looser (group lasso) construction strategies were unable to achieve promising outcomes.

## Author contributions

This manuscript has not been published or presented elsewhere in part or in entirety, and is not under consideration by any another journal. This study was approved by the medical ethics committee of Shanxi Province, and the approved certification number is 2012013. All subjects have been given written informed consent in accordance with the Declaration of Helsinki. Meanwhile, all the authors have read through the manuscript, approved it for publication, and declared no conflict of interest. JC had full access to all of the data in the study and takes responsibility for its integrity and the accuracy of data analysis.

### Conflict of interest statement

The authors declare that the research was conducted in the absence of any commercial or financial relationships that could be construed as a potential conflict of interest.
