# Supplementary material for: Resting-State Brain Functional Hyper-Network Construction Based on Elastic Net and Group Lasso Methods
Source: Front Neuroinform. 2018 May 15;12:25. doi: 10.3389/fninf.2018.00025 (PMC5962886; doi:10.3389/fninf.2018.00025)
Supplement: Supplementary file 2 [file Presentation_2.pdf]

Supplemental Text S2. Results of multiple linear regression analysis between network properties and confounding variables.

Confounding Variables	Coefficients	Std. Error	T Stat.	P-value	Lower 95%	Upper 95%
lasso						
Clustering Coefficient HCC[1] (Adj. R_{sqr} = -0.037, P = 0.878)						
Intercept	1.308	0.046	28.412	<0.001	1.216	1.400
Gender	-0.010	0.017	-0.582	0.563	-0.045	0.025
Age	0.001	0.001	0.651	0.517	-0.001	0.002
Educational Attainments	0.000	0.007	0.052	0.958	-0.012	0.013
Clustering Coefficient HCC[2] (Adj. R_{sqr} = 0.021, P = 0.230)						
Intercept	0.838	0.106	7.929	<0.001	0.627	1.049
Gender	0.061	0.040	1.516	0.134	-0.019	0.141
Age	0.001	0.002	0.461	0.646	-0.003	0.005
Educational Attainments	0.016	0.015	1.066	0.291	-0.014	0.046
Clustering Coefficient HCC[3] (Adj. R_{sqr} = -0.003, P = 0.434)						
Intercept	0.310	0.057	5.439	<0.001	0.196	0.424
Gender	0.028	0.022	1.296	0.200	-0.015	0.071
Age	$-5.625E^{-5}$	0.001	-0.051	0.960	-0.002	0.002
Educational Attainments	0.007	0.008	0.817	0.417	-0.010	0.023
elastic_net						
Clustering Coefficient HCC[1] (Adj. R_{sqr} = 0.067, P = 0.163)						
Intercept	1.472	0.034	43.348	<0.001	1.404	1.539
Gender	-0.030	0.013	-1.651	0.122	-0.056	-0.004
Age	$6.686E^{-5}$	0.001	0.101	0.920	-0.001	0.001
Educational Attainments	-0.005	0.005	-1.066	0.290	-0.015	0.005
Clustering Coefficient HCC[2] (Adj. R_{sqr} = -0.016, P = 0.577)						
Intercept	0.981	0.092	10.627	<0.001	0.797	1.166
Gender	0.035	0.035	1.012	0.316	-0.035	0.105
Age	0.001	0.002	0.486	0.629	-0.003	0.004
Educational Attainments	0.008	0.013	0.618	0.539	-0.018	0.034
Clustering Coefficient HCC[3] (Adj. R_{sqr} = -0.014, P = 0.557)						
Intercept	0.310	0.042	7.430	<0.001	0.226	0.393
Gender	0.022	0.016	1.363	0.178	-0.010	0.053
Age	$5.548E^{-5}$	0.001	0.068	0.946	-0.002	0.001
Educational Attainments	0.001	0.006	0.198	0.844	-0.011	0.013
group_lasso						
Clustering Coefficient HCC[1] (Adj. R_{sqr} = 0.062, P = 0.174)						
Intercept	1.626	0.077	21.014	<0.001	1.471	1.780
Gender	-0.014	0.029	-0.478	0.635	-0.073	0.045
Age	-0.004	0.002	-1.682	0.116	-0.007	0.001
Educational Attainments	-0.008	0.011	-0.738	0.463	-0.030	0.014
Clustering Coefficient HCC[2] (Adj. R_{sqr} = 0.146, P = 0.205)						

Intercept	1.234	0.111	11.114	<0.001	1.012	1.456
Gender	0.007	0.042	0.166	0.869	-0.077	0.091
Age	0.008	0.002	1.392	0.124	0.004	0.012
Educational Attainments	0.001	0.016	0.039	0.969	-0.030	0.032
Clustering Coefficient HCC[3] (Adj. R_{sqr} = -0.009, P = 0.492)						
Intercept	0.428	0.087	4.939	<0.001	0.255	0.601
Gender	0.039	0.033	1.188	0.239	-0.027	0.105
Age	$2.366E^{-6}$	0.002	0.001	0.999	-0.003	0.003
Educational Attainments	0.010	0.012	0.799	0.427	-0.015	0.034

The range of age is 17–51 years. Optional values of gender are male and female. Optional values of educational attainments are illiteracy, primary school, junior high school, senior high school, junior college, college, graduate degree and above. Adj. R_{sqr}, adjusted R square.Coefficients, regression coefficient. Std. Error, standard error. T stat., T statistic. Lower 95%, low bound of 95% confidence limits. Upper 95%, upper bound of 95% confidence limits.
